# *Enterococcus faecium* are associated with the modification of gut microbiota and shrimp post-larvae survival

**DOI:** 10.1186/s42523-021-00152-x

**Published:** 2021-12-24

**Authors:** Shicong Du, Wei Chen, Zhiyuan Yao, Xiaolin Huang, Chen Chen, Haipeng Guo, Demin Zhang

**Affiliations:** 1 State Key Laboratory for Managing Biotic and Chemical Threats To the Quality and Safety of Agro-Products, Ningbo University, Ningbo, 315211 China; 2 School of Marine Sciences, Ningbo University, Ningbo, 315211 China; 3 School of Energy and Environment, City University of Hong Kong, Hong Kong, SAR China; 4 School of Civil and Environmental Engineering, Ningbo University, Ningbo, 315211 China; 5 Zhejiang Mariculture Research Institute, Wenzhou, 325099 China

**Keywords:** Shrimp post-larvae, Survival, *Enterococcus faecium*, Gut microbiota, Predicted function

## Abstract

**Background:**

Probiotics are widely used to promote host health. Compared to mammals and terrestrial invertebrates, little is known the role of probiotics in aquatic invertebrates. In this study, eighteen tanks with eight hundred of shrimp post-larvae individuals each were randomly grouped into three groups, one is shrimps administered with *E. faecium* as probiotic (Tre) and others are shrimps without probiotic-treatment (CK1: blank control, CK2: medium control). We investigated the correlations between a kind of commercial *Enterococcus faecium* (*E. faecium*) powder and microbiota composition with function potentials in shrimp post-larvae gut.

**Results:**

We sequenced the 16S rRNA gene (V4) of gut samples to assess diversity and composition of the shrimp gut microbiome and used differential abundance and Tax4Fun2 analyses to identify the differences of taxonomy and predicted function between different treatment groups. The ingested probiotic bacteria (*E. faecium*) were tracked in gut microbiota of Tre and the shrimps here showed the best growth performance especially in survival ratio (SR). The distribution of SR across samples was similar to that in PCoA plot based on Bray-Curits and two subgroups generated (SL: SR < 70%, SH: SR ≥ 70%). The gut microbiota structure and predicted function were correlated with both treatment and SR, and SR was a far more important factor driving taxonomic and functional differences than treatment. Both Tre and SH showed a low and uneven community species and shorted phylogenetic distance. We detected a shift in composition profile at phylum and genus level and further identified ten OTUs as relevant taxa that both closely associated with treatment and SR. The partial least squares path model further supported the important role of relevant taxa related to shrimp survival ratio.

**Conclusions:**

Overall, we found gut microbiota correlated to both shrimp survival and ingested probiotic bacteria (*E. faecium*). These correlations should not be dismissed without merit and will uncover a promising strategy for developing novel probiotics through certain consortium of gut microbiota.

**Supplementary Information:**

The online version contains supplementary material available at 10.1186/s42523-021-00152-x.

## Introduction

The gut microbiota has emerged as a key regulator of host health, affecting digestion, immune activities, and homeostasis maintenance [1–4]. As such, the characterization and subsequent manipulation of this microscopic community is a supportive proposition for aquaculture research. *Litopenaeus vannamei*, a valuable and favored specie for many Asian countries [5], has subsequently dominated the field of aquatic invertebrate gut microbiomes [6]. With increasingly threatened by the onset of various diseases in shrimp aquaculture [7], the most common interventions, such as antibiotics [8, 9] and probiotics [10, 11], have been conducted to produce numbers of possible positive effects on the shrimp host. Considering the pernicious side effects of antibiotics and other chemotherapeutic agents, such as antibiotic resistance and the production of acute toxic to aquatic organisms [8], probiotics have become promising candidates for maintaining host health for its “green” and long-term research [6]. However, in comparison with mammals and terrestrial invertebrates, relatively very little is known about how probiotics impact gut microbiota of aquatic invertebrates.

It has been recognized that probiotics can increase competition, potentially supporting colonization resistance against pathogens in the gut, and thereby to promote shrimp survival and other growth performance [12]. For instance, shrimps treated with *Lactobacillus. plantarum* in their diet showed a significant increase in survival rate and growth, along with a reduction of *Vibrio. harvey* that is a common cause of shrimp mortalities [10]. The probiotic consortium has demonstrated an effective way to reduce the abundance of pathogenic *Vibrio* species and to prevent the mortality during *Vibrio* challenges [13]. Such studies on shrimp have mainly focused on the physiological and immunological responses to the applied probiotics. Even there were a range of probiotics showed unstable effects or they were not beneficial to the host [14, 15]. Most importantly, probiotics must be able to pass through the gut and information about whether the ingested probiotics can be detected in the gut microbiota remains scarce and contradictory [16, 17]. To increase resolution and enhance the likelihood of identifying ecotypes of ingested probiotic bacteria, a novel supervised computational pipeline, named oligotyping pipeline, has been further conducted [18].

Previous studies in human model focusing on the microecological effects of probiotics on gut microbiota were conflicting. Some studies disclosed that probiotics may perturb the function of indigenous microbiota [19, 20], whereas Kristensen et al. [21] argued that probiotics may promote the homeostasis of the gut microbiota without altering its composition. Regarding to shrimp, it is often suggested that a reduction in bacterial diversity (Shannon and Richness) within the gut or the differential abundance of certain bacteria may be responsible for the onset of pathogenesis [22, 23]. However, lower species diversity was also in healthy one [24]. Since many debates over these still exist and it is often impossible to discern between cause and effect, related research is still going on. Furthermore, the significance of shaping gut microbiome in larvae stage or early has been recognized recently [25]. The initial colonizers of the gut microbiota may act as a natural barrier to subsequent bacterial colonization and thus have long-term effects on host development [22, 26]. However, several probiotic species that are not indigenous to the marine environment with limited proliferation potential are known to be bad colonizers [27]. Given the host-specific gut microbiota, exploration of its composition and function potentials coupled with administrated probiotic bacteria will extend our knowledge of their relationships in how to regulate host health.

Modular co-occurrence network has been used to provide novel insights into potential interactions by revealing the niche spaces shared by modular members [28]. The clustering of microbial species into distinct modules has been used to infer gathering mechanism [29, 30]. In this study, we used 16S rRNA gene (V4 region) amplicon sequencing and Tax4Fun2 with six biological replicate tanks to measure gut microbiota and predicted function of post-larval shrimp. We compared the difference of gut microbiota composition and function potentials between shrimps administered with *Enterococcus faecium* as probiotic and those without probiotic-treatment, and thereby to address three key questions: (i) Can administrated probiotics be identified in the shrimp gut after 51-day continuous feeding? (ii) How does this probiotic impact the gut microbiota composition and predicted function? (iii) What are the relationships between gut microbiota and shrimp survival?

## Results

### Shrimp performance and environmental factors

During shrimp culture, there were no significant variation in environmental factors, such as water temperature, dissolved oxygen (DO), pH, ammonium, and nitrite across all groups (Additional file 1: Fig. S1). At the end of cultivation, we calculated growth performance of all survivors each tank (Additional file 1: Table S1). The survival ratio (SR) is recognized as a crucial indicator for shrimp in post-larvae or before. We observed that the survival ratio of shrimp with probiotic administration (Tre) was significantly higher than that of the shrimp without probiotic administration (CK1, blank control with additive-free feed; CK2, medium control with medium-treated feed) (Table 1, *P* < 0.05). Notably, a larger difference of survival ratio was found within shrimps of the controls, while shrimps of Tre showed a more consistent survival pattern (Additional file 1: Fig. S2). The other indexes characterizing shrimp growth performance, such as unit yield (UY) and specific growth ratio (SGR) of shrimp was also higher in Tre. Among these, UY of Tre was significantly higher than CK2 (*P* < 0.05), and FCR of Tre was significantly lower than CK2 (*P* < 0.05), but both were non-significant compared to CK1 (Table 1).

**Table 1:** Survival ratio (SR), unit yield (UY), and specific growth ratio (SGR) of shrimp and the feed conversion ratio (FCR) after 51 days

	CK1	CK2	Tre	*P*
SR (%)	65.33 ± 14.10b	58.66 ± 24.89b	84.33 ± 9.03a	**0.032**
UY (g/m^3^)	154.62 ± 42.81ab	133.42 ± 56.32b	196.00 ± 20.37a	0.054
SGR	8.66 ± 1.65	7.66 ± 0.60	8.72 ± 1.17	0.313
FCR	0.97 ± 0.19ab	1.09 ± 0.25b	0.82 ± 0.08a	**0.032**

The data represents the mean ± standard deviation (n = 6). Survival ratio (SR) equals the number of surviving shrimps divided by initial number (number of surviving shrimp number was calculated by total weight and individual weight); Unit yield (UR) means weight of shrimp per unit of water volume; Specific growth ratio (SGR) is the ratio of growth rate to days of growth; feed conversion ratio (FCR) was calculated as the number of kilograms of feed that are used to produce one kilogram of whole shrimp. Different lower-case letters represent differences between the two groups that were significant at *P* < 0.05 according to Mann–Whitney U test. Statistically significant *P* estimated by Krustkal–Wallis H test is shown in bold

### Tracking probiotic bacteria in gut microbiota

**Fig. 1:**
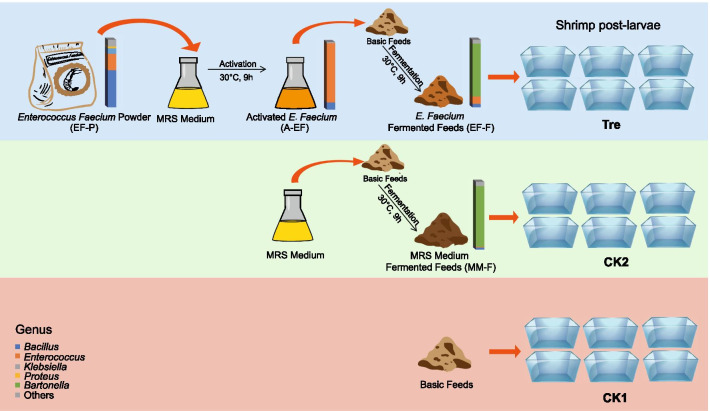
Experimental design and the dynamic compositional variation of *Enterococcus faecium* powder (EF-P) (bar chart, abundant (average relative abundance > 1%) genera in EFP)

In order to increase the amount of effective probiotic bacteria, activation is the regular step before its application into aquaculture [14]. After activation, the relative abundance of *Enterococcus* significantly increased from 24.29% in *E. faecium* powder (EF-P) to 87.42% in activated *E. faecium* (A-EF), and then sharply dropped to 10.85% in *E. faecium*-fermented feeds (EF-F) (Fig. 1, Additional file 1: Table S2). After 51 days, *Enterococcus*, which accounted for 0.5% relative abundance of all samples, was the only genus from EF-P that could be traced in the Tre gut. Oligotyping pipeline [18] was conducted to finely track the source of *Enterococcus* in the gut microbiota*.* The OTUs were limited to those annotated as *Enterococcus* in our dataset. Two information-rich positions of the aligned 16S rRNA V4 sequences were identified by Shannon entropy analysis. Overall, *Enterococcus* comprised 12 major oligotypes that were almost exclusively present in gut samples of Tre and EF-P samples (Additional file 1: Fig. S3). Up to 92% of these sequences were represented by OTU 1462 (*Enterococcus*), which was identified as *E. faecium* except oligotype “CT” from the EzBioCloud database [31]. Moreover, OTU1462 was the most abundant taxa affiliated to *Enterococcus* in EF-P (71.19%), A-EF (86.36%), and EF-F (82.85%), and it was mostly found in gut microbiota of Tre (Additional file 1: Fig. S4).

### The diversity analysis of Gut microbiota composition

**Fig. 2:**
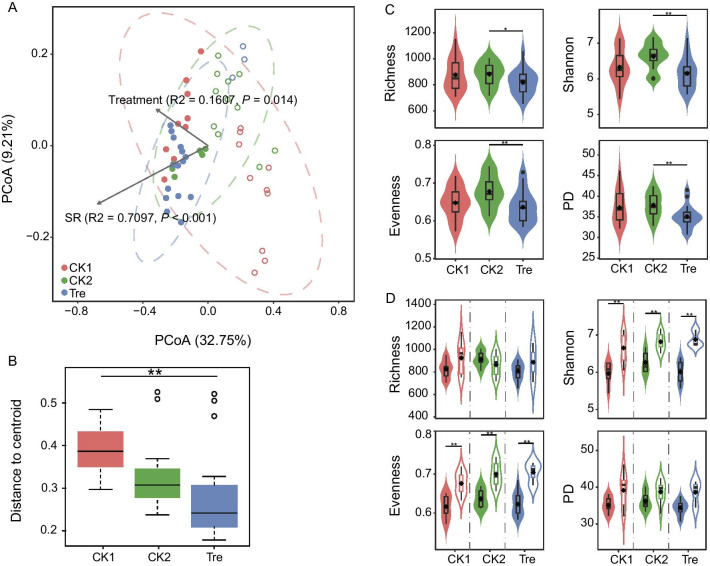
Diversity of gut microbiota. **A** Principal coordinates analysis plots of gut microbiota Bray–Curtis beta diversity for treatment and survival ratio (SR). The dotted ellipses with corresponding color for treatment represent the 95% confidence interval. The hollow circle represents shrimp survival ratios of ≤ 70% (SL), while the solid circle represents shrimp survival ratios of > 70% (SH). **B** Boxplot of Bray–Curtis distance between samples and group centroids (**P < 0.01, Tukey's 'Honest Significant Difference'). **C** Alpha-diversity indexes of gut microbiota for treatment (*P < 0.05, **P < 0.01, Mann–Whitney U test). **D** Alpha-diversity indexes of gut microbiota for SR in each treatment group. The hollow represents SL, while the solid represents SH (*P < 0.05; **P < 0.01; Mann–Whitney U test)

Principal coordinate analysis (PCoA) based on Bray–Curtis distance was carried out to study the effect of treatment on the gut microbiota structure (Fig. 2A), and objects driven by treatment didn’t exhibit evident clusters. However, there was a shorter distance from objects to centroid in Tre compared with the controls (Fig. 2B), which was supported by ANOSIM analysis that showed samples outside Tre group are more different than those within Tre group (Table 2). Unexpectedly, a clearly more-consistent cluster with 32.75% explained variance at the fist dimension was observed (Fig. 2A). We tried to map these objects to SR for their similar separation pattern of samples (Additional file 1: Fig. S2), and thus two subgroups bounded by 70% SR were generated; one is tanks with less survivors (SL, SR < 70% with total 24 samples), another is tanks with more survivors (SH, SR ≥ 70% with total 30 samples). Changes in the gut microbiota structure occurred between SL and SH were also supported by ANOSIM (Table 2). Furthermore, a PERMANOVA test was applied to identify the most important factors driving the structure differences between gut microbiomes. The results identified that treatment combined with SR (F test = 7.28, R^2^ = 0.43, *P* < 0.001) explained 43% of the between-sample variance. Specifically, SR (F test = 3.40, *R*^2^ = 0.12, *P* < 0.001) was a more important factor driving the structure differences than treatment (F test = 19.33, *R*^2^ = 0.27, *P* < 0.001). Meantime, vectors were loaded to estimate the contribution of two factors, and the result also emphasized that SR contributes far more to the gut microbiota structure than treatment (Fig. 2A).

**Table 2:** Analysis of similarity (ANOSIM) of the difference in gut microbiota grouped by treatment and the survival ratio for each group, based on Bray–Curtis distance

Grouped by treatment	R	*P*
Global	0.140	**0.002**
CK1 versus CK2	0.047	0.109
CK1 versus Tre	0.171	**0.005**
CK2 versus Tre	0.221	**0.001**

Grouped by survival ratio (SH vs. SL)	R	*P*
Global	0.579	**0.001**
CK1	0.898	**0.001**
CK2	0.403	**0.003**
Tre	0.991	**0.001**

Statistically significant values at *P* < 0.05 are shown in bold

Regarding to bacterial α-diversity, the community of Tre showed lower values than that observed in the controls (Fig. 2C), indicating a less species, more similar taxonomy, albeit uneven species in the gut microbiota of Tre. However, significant differences (*P* < 0.05) were only observed between Tre and CK2, not between Tre and CK1 (Fig. 2C). For grouping by SR in each group, only CK2 showed a slightly higher community species in SH compared with that observed in SL (Fig. 2D). Both Shannon and Evenness indexes showed significantly lower values in SL than that of SH (*P* < 0.01). Although not statistically, the values of PD also showed a certain difference between SH and SL (Fig. 2D). In addition, the community-level habitat niche breadths were further estimated to determine the range of resources a species uses. A relatively wider niche was observed in the gut microbiota of Tre (Additional file 1: Fig. S5A), but they were non-significant. The gut microbiota of SH exhibited significantly higher niche breadth values than that of SL (*P* < 0.001) (Additional file 1: Fig. S5B).

### The difference in community composition among shrimp categories

The distribution of the main bacterial phyla/genera characterizing the gut microbiota were related to treatment and SR in the study (Additional file 1: Fig. S6). Compared with two control groups, Proteobacteria (CK1: 35.70%, CK2: 37.31, Tre: 27.09%) was subjected to a significant reduction in the gut microbiota of Tre where Bacteroides (CK1: 26.55%, CK2: 25.51%, Tre: 34.44%) accounted a major proportion (Additional file 1: Table S3 and Fig. S6A). Regarding to SR subgroups, the major phyla distribution in the gut microbiota of SH were the same as that of Tre (Additional file 1: Table S3 and Fig. S6A). Besides, the relative abundances of Planctomycetes, Chloroflexi, Tenericutes were significantly higher in SL compared with SH, whereas Verrucomicrobia and Saccharibacteria demonstrated an opposite trend (*P* < 0.05, Additional file 1: Table S3 and Fig. S6A).

At the genus level, the relative abundance of *Algoriphagus* was more differentially abundant in gut microbiota of Tre than that of two control groups while *Vibrio* was observed enriched in gut microbiota of CK1 and CK2 (Additional file 1: Table S3 and Fig. S6B). For grouping by SR, the relative abundances of *Planctomyces*, *Haliea*, *Vibrio*, *Mycobacterium*, *Rhodopirellula*, *Ruegeria*, *Winogradskyella*, *Candidatus Bacilloplasma*, and *Nitrosomona* were significantly higher in SL compared with that of SH. On the contrary, the gut microbiota of SH significantly enriched *Haloferula*, *Formosa*, *Algoriphagus* and *Pirellula* (*P* < 0.05, Additional file 1: Table S3 and Fig. S6B).

**Fig. 3:**
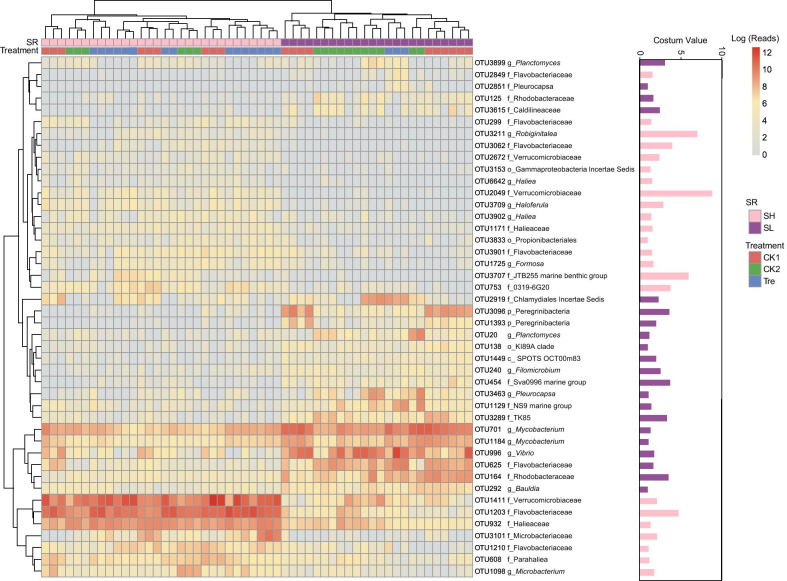
Heatmap of differentially abundant OTUs for shrimp survival (SR) using ANCOM with custom value. The purple bar represents the OTUs were more prevalent in the gut of shrimp who survived less well, whereas the pink bar represents OTUs were more prevalent in the gut of shrimp who survived well

**Fig. 4:**
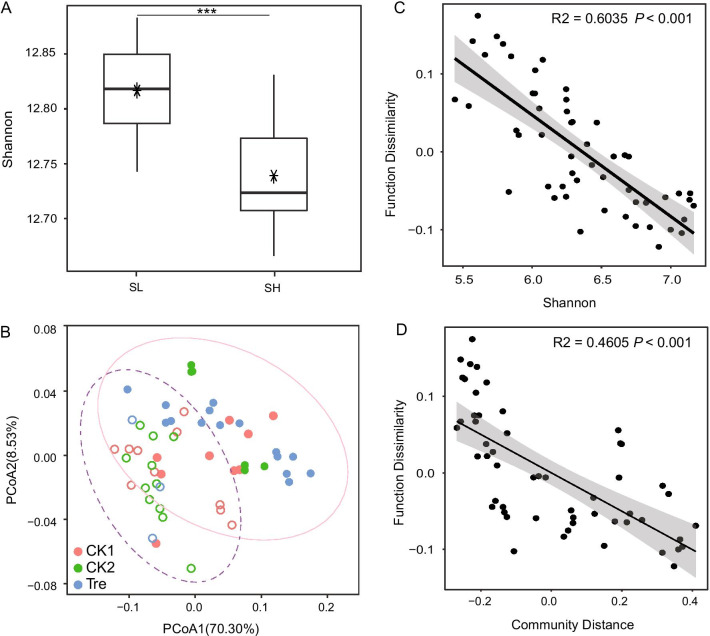
Diversity of predicted function (Kos) in gut microbiome. **A** Alpha diversity (Shannon) of predicted function in gut microbiota (* *P* < 0.05; ** *P* < 0.01; Mann–Whitney U test). **B** Principal coordinates analysis plots of predicted function Bray–Curtis beta diversity for treatment and survival ratio (SR). The dotted ellipse border for SL and the solid ellipse border for SH both represent the 95% confidence interval. The hollow circle represents shrimp survival ratios of ≤ 70% (SL), while the solid circle represents shrimp survival ratios of > 70% (SH). **C** Relationships between function dissimilarity and Shannon in gut microbiota. **D** Relationships between function dissimilarity and community distance in gut microbiota

Differentially abundant OTUs were further identified by ANCOM analysis (Additional file 1: Fig. S7). OTU1462 was recognized as the applied probiotic bacteria according to oligotyping pipeline and was enriched in gut microbiota of Tre (Additional file 1: Fig. S4). Corresponding to this result, several OTUs including OTU1203 (Flavobacteriaceae), OTU996 (*Vibrio*), OTU1462 (*Enterococcus*), etc. were observed enriched in gut microbiota of Tre. However, it is worth mentioning that higher relative abundance of OTU996 (*Vibrio*) here was mainly attributed to gut samples from low-survival shrimp that contributed 95.13% (Additional file 1: Fig. S7). Significantly less abundant OTUs, for instance, OTU1189 (*Vibrio*), OTU879 (Phyllobactereriaceae) and OTU368 (*Erythrobacter*) were observed in gut microbiota of Tre (Additional file 1: Fig. S7). Further, ANCOM analysis identified 44 differentially abundant OTUs based on SR (Fig. 3). Among these, 23 OTUs with higher abundance in SH were mainly members of families Flavobacteriaceae (8/23), Halieaceae (5/23), and Verrucomicrobiaceae (4/23). However, the taxon distribution of 21 OTUs with higher abundances in SL was relatively more dispersed and was mainly attributed to families Planctomycetaceae (2/21), Rhodobacteraceae (2/21), and Microbacteriaceae (2/21). Among them, two potential pathogens, OTU996 (*Vibrio*) and OTU1469 (*Chlamydiales Incertae Sedis*) were found (Fig. 4).

### Predicted functions of gut microbiota

**Fig. 5:**
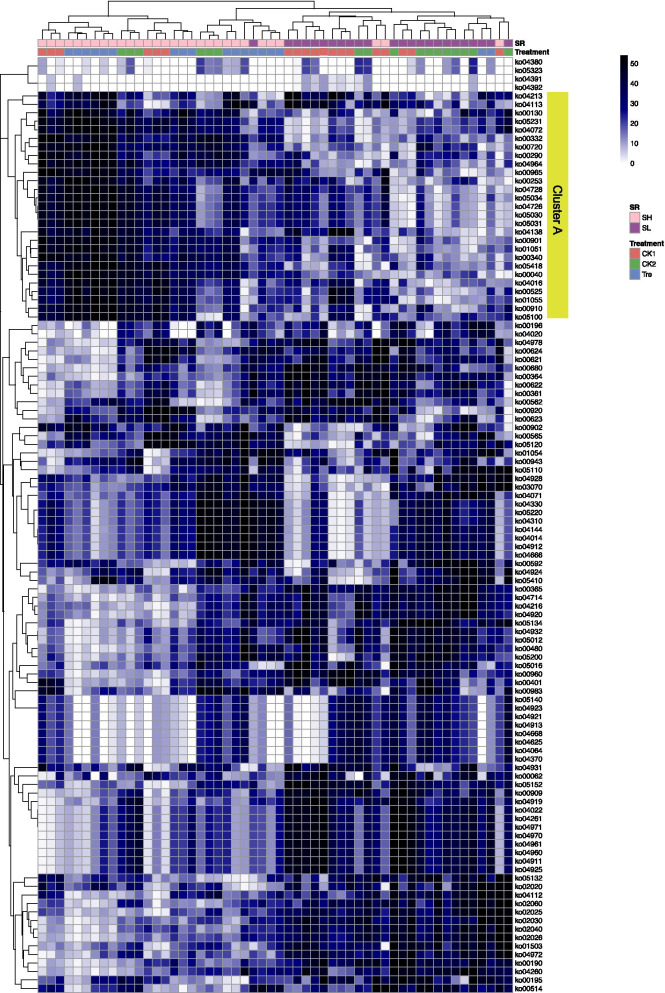
Heatmap of differentially abundant Kos and the relative abundance of Ko was normalized by “range”

Tax4Fun2 prediction was applied to assess how treatment and SR affect the potential function and pathway of gut microbiota and 7843 Kos generated based on 3553 OTUs. Unexpectedly, treatment didn’t shape the within-sample diversity (Shannon) of predicted function (*P* > 0.05). Otherwise, the values of Shannon index showed a significant difference between SL and SH (*P* < 0.05, Fig. 4A), which supported by PCoA where objects grouped by SR showed an almost clear separation based on Bray–Curtis distance (Fig. 4B). Consistently, PERMANOVA indicated that treatment and SR explained 9.04% and 28.63 variances in predicted function, respectively. In addition, function dissimilarity presented significant correlation with Shannon index (R^2^ = 0.6035, *P* < 0.001) and community distance (R^2^ = 0.4605, *P* < 0.001), indicating the more species, the more similar the predicted function composition. Furthermore, 110 significantly abundant pathways (organismal systems, cellular process, metabolism, etc.) were identified using Monte Carlo multiple randomized approach at 95% confidence interval (Fig. 5). The data was standardized by “range” and showed a clear cluster enriched in SH (Cluster A) (Additional file 2). Regarding to metabolism in cluster A, these were mainly involved amino acid metabolism, biosynthesis of other secondary metabolites, energy metabolism and metabolism of terpenoids and polyketides (Additional file 2). These results indicated that close interconnection between predicted function and gut microbiota.

### Ecological network analysis on the gut microbiota

A metacommunity network was constructed to estimate the bacterial interaction based on Spearman’s rank correlation (Additional file 1: Fig. S8). The entire network was modularized, and top eight modules (with 65.7% node coverage) were studied (Additional file 1: Fig. S8A). Out of the total significant co-occurrent features, 17.96% were allocated in module I, 15.29 in module II, etc. (Fig. S8B). Most modules were almost specific (relatively more abundant) to a single group, either to two control groups (Additional file 1: Fig. S8B). In addition, OTUs from module I were mainly affiliated to families Rhodobacteraceae, Vibrionaceae, and Microbacteriaceae, whereas OTUs from module II were primarily affiliated to families Halieaceae, Flavobacteriaceae, and Verrucomicrobiaceae.

**Fig. 6:**
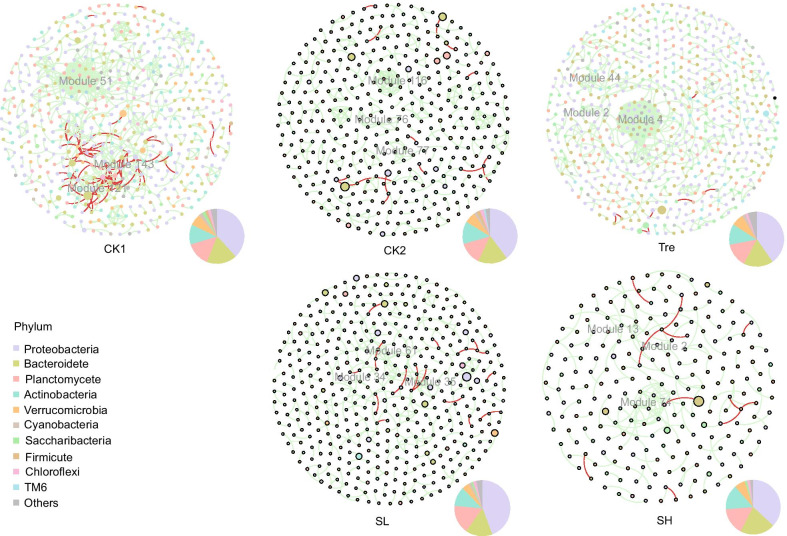
Co-occurrence patterns of gut microbiota for subgroup. There are 156, 159, 152, 149 and 82 modules in CK1, CK2, Tre, SL and SH, respectively. The top 3 modules with more OTUs are marked in each network. The size of each node is proportional to the number of relative abundances. The nodes are colored according to phylum

The network for each subgroup was separately generated to explore their specific co-occurrence pattern (Fig. 6). The topological properties of each subgroup showed more diverse co-occurrence pattern in treatment group while SL and SH showed a more similar pattern (Fig. 6, Additional file 1: Fig. S9, Table S5). Specifically, the network of Tre showed the highest values of degree and eigenvector centrality compared to the two control groups. The network of CK2 showed the highest value of betweenness centrality and extremely low value of closeness centrality (Additional file 1: Fig. S9A). For SR subgroup, the nodes in network of SH with significantly higher closeness centrality and lower eigenvector centrality compared with that of SL (Additional file 1: Fig. S9B). Considering the high level of modularity in these network (Additional file 1: Table S5), we further analyzed the taxonomy of nodes in top three modules that account for 19.29%, 6.41%, 16.47%, 12.46% and 20.27% in the network of CK1, CK2, Tre, SL and SH, respectively (Additional file 3). OTUs from module 51, 143, 121 in CK1 were mainly affiliated to families Planctomycetaceae, Rhodobacteraceae, Flavobacteriaceae, and OTUs from module 77, 116, 76 in CK2 mainly affiliated to families Flavobacteriaceae, Halieaceae. As for Tre, OTUs from module 4, 2, 44 mainly affiliated to families Marinilabiaceae, Vibrionaceae, Rhodobacteraceae. In SL, OTUs from module 61, 35, 34 mainly affiliated to families Rhodobacteraceae, Planctomycetaceae, while most of the OTUs affiliated to family Flavobacteriaceae in SH (Additional file 3).

### Relevant taxa related to shrimp survival

**Fig. 7:**
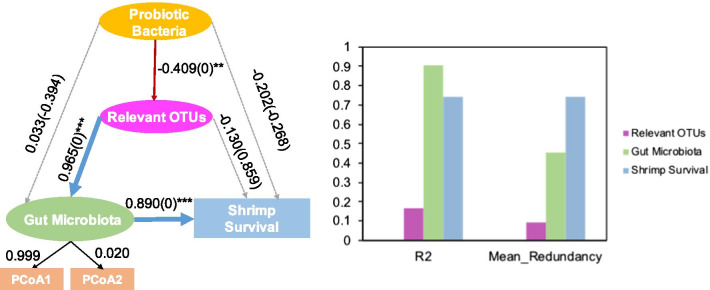
The final path of partial least squares path model (PLS-PM) quantification of the direct and indirect effects of probiotic bacteria and relevant OTUs on gut microbiota, based on Bray–Curtis distance and shrimp survival. Blue and red lines represent positive and negative effects, respectively. Dashed lines indicate the coefficients that did not significantly (*P* > 0.05) differ from 0. The wider the line, the higher the absolute value of the coefficient obtained with PLS-PM. (** *P* < 0.01; *** *P* < 0.001)

Using Boruta analysis, 10 OTUs that were possibly related to probiotics and shrimp survival were identified and defined as relevant OTUs (Additional file 4). To better analyze the relationship among applied probiotic bacteria (OTU 1462), relevant OTUs, gut microbiota, and shrimp survival, a partial least squares path model (PLS-PM) was constructed. This model was assessed using the Goodness of Fit (GoF) statistic, and the GoF value of which was 0.534. Unexpectedly, probiotic bacteria exerted a direct and weak association with gut microbiota (0.033) and shrimp survival (− 0.202) but showed a strong association with relevant OTUs (− 0.409) (Fig. 7). Relevant OTUs showed a strong and direct association with gut microbiota (0.965) and exhibited a non-significant association with shrimp survival (0.729) with a combination of direct (− 0.130) and indirect (0.859) associations. As expected, gut microbiota exerted a significant and direct association with shrimp survival (0.890) (Fig. 7). In this model, the average redundancy for shrimp survival represents that probiotic bacteria, relevant OTUs and gut microbiota predict 74% of the variability of survival indicator (Fig. 7).

## Discussion

*Enterococcus faecium* powder, a popular probiotic agent in aquaculture, was used to study the correlations between probiotics and gut microbiota. In practice, probiotics are activated to increase the number of effective bacteria before application. In this study, the relative abundance of *Enterococcus* greatly varied after activation and a 9-h fermentation with feeds (Fig. 1). The effects of feed fermentation on the gut microbial community varied with substrate, and inappropriate methods may counteract the beneficial effects of probiotics [14, 32]. Although not all of them require such operation, more attention should be paid to probiotic fermentation method especially the specific requirements and appropriate culturing conditions of target probiotics [33].

Although we could not determine whether *E. faecium* would be a good gut colonizer without supported result from Fluorescence in situ Hybridization (FISH), it was indeed tracked in gut microbiota with a total 0.5% abundance. Previous studies suggested that probiotic *E. faecium* could be isolated from healthy shrimps with antibacterial and adhesive activities [34, 35]. Although stable colonization is not necessarily required for probiotics to be beneficial to host health, a substantial population must, at least transiently, establish for them to have a metabolic impact on the host and the indigenous gut microbiota [36]. However, the strong correlation between probiotic and shrimp survival along with a small within-group difference in Tre was observed (Tables 1 and 2, Additional file 1: Fig. S2). One possible supported explanation for this is the critical role of rare species in regulating the bacterial community, which has been widely demonstrated in various natural ecosystems [37, 38] and host-associated microbiomes [39]. Notably, it may vary greatly depending on the niche and the species that is introduced, the time it is analyzed and the immune response in case of animals. In addition, the shrimp post-larvae used in this study may contribute to the prominent effect of such few proportions of *Enterococcus.* The development of microbiota within the gut is reported to be influenced by priority effects, in which the order and timing of species arrival determine how the species affect each other [40]. Previous studies proposed that probiotics may show a weaker effect on older than younger individuals [41–43]. Hence, we inferred that probiotic administration should be adopted sufficiently early to achieve the optimal effect and further study are required to support it.

Changes to the gut microbiota have been implicated in a wide range of host health in humans [44], as well as in shrimps [2, 22, 23, 45]. Specifically, the “healthy” gut microbiota was often characterized by high species diversity and function redundancy, less potential pathogens. The correlations between “healthy” gut microbiota and these “healthy” features should not be dismissed without merit and it revealed a general pattern in kinds of animals. However, diversity is the question, not the answer [46]. In our study, gut microbiota of Tre and SH indicated a simplified species community (lower values of Shannon and Richness) with a cohort of dominant taxa (lower value of Evenness) (Fig. 2C, D), which was also observed in gut microbiota of probiotic-treated fish [47]. The predicted function of a metacommunity appeared to be more unitary relative to the diverse community species (Fig. 4C, D), indicating the whole community may exist stronger function redundancy. In that case, we can infer that even if the species diversity of the gut microbiota of Tre and SH is low, their function potentials may be sufficient to maintain the whole ecosystem. Some predicted metabolic pathways, such as amino acid metabolism and energy metabolism, were enriched in SH group (Fig. 5), playing a key role in energy homeostasis [48]. In addition, it also can be well supported by a homogeneous composition structure (Fig. 2B) and a shorter phylogenetic distance (Fig. 2C, D) that were observed in Tre and SH group, suggesting that close microbial lineages may display ecological similarities, potentially accounting for the community cluster [49, 50].

Most pathogens that impact aquaculture are deemed opportunistic, including *Vibrio*. Previous studies have reported that was closely related to the onset of various diseases and mass mortality of shrimp [10, 11, 13]. As expected, we observed significantly abundant *Vibrio* in gut microbiota of shrimps who did not survive as well (Additional file 1: Table S3). However, those shrimps in SL with more abundant *Vibrio* were still survivors, that is to say, *Vibrio* strains can co-exist with other bacteria even with those ingested probiotic bacteria (Additional file 1: Fig. S7). Once dysbiosis in aquaculture, they will apparently exploit chance to emerge as virulent and damaging [51]. In that case, the antibacterial activity of *Enterococcus* strains against *Vibrio* may be shown [35, 52], which was supported by the result of this study that *Vibrio* was less abundant in gut microbiota of probiotic-treated shrimp (Additional file 1: Table S3). Otherwise, the modularity of ecological networks is thus essential to uncover how microbiome assembly influences microbiome structure [53]. The module 4 with highly modulization in gut microbiota of Tre showed members of genus *Vibrio* were more co-occurred with other taxa (Fig. 6, Additional file 3), which could infer that probiotic was associated with the more interactions occurred between certain taxa and *Vibrio* species*.* Besides, those centrality metrics of network grouped by treatment seemed more closely related to total graph sizes, for example, closeness is essentially a global centrality version of degree [30]. Gut microbiota of Tre showed a more co-occurrence within the community, which can be inferred that there are more underlying interactions that associated with probiotics.

Finally, we identified 10 relevant OTUs that were differentially abundant and associated with surviving individuals (Additional file 4). Among them, some members of Flavobacteriaceae are known for their ability to degrade and metabolize refractory organic compounds [54]. Certain planktonic Verrucomicrobiaceae phylotypes have the potential to be active polysaccharide degraders [55, 56], promoting food utilization to achieve enhanced growth performance. Additionally, several genera of Verrucomicrobiaceae have been isolated from marine animals, which might be closely related to the host health. Halieaceae, a newly found family, has been reported its metabolic capabilities, such as anoxygenic photosynthesis [57]. However, the PLS-PM model showed that these relevant OTUs strongly correlated with applied probiotic and the modification of gut microbiota and shrimp health (Fig. 7). Large-scale studies have demonstrated that probiotics indeed cause shifts in the compositions of gut microbiota [14, 58, 59]. However, few reports have proposed that probiotics play growth-promoting roles in shrimps in collaboration with gut “partners” in shrimps. This relationship may explain why multi-strain probiotics appear to show greater efficacy than single‐strain probiotics [60]. Notably, uncultured bacteria accounted for a considerable proportion of the indicators in this work (Additional file 4), which may indicate that they play a vital role in host health, with unexplored function. In the future, it will be more conducive to accurately screen host-specific taxa at the strain level. Deeper verification tests based on metagenomics and culturomics are necessary, which will be further applied in the probiotic field and provide insights into the relationship between interspecies interaction and shrimp survival.

## Materials and methods

### Experimental design and sample collection

The post-larval shrimp (*Litopenaeus vannamei*) tanks in this study are located in Zhejiang Mariculture Research Institute, Wenzhou, China (27° 51′ N, 120° 50′ E). All test tanks were equal in size (600 L) with 400 L aquaculture water. Both the daily water exchange rates and feed amount (Xiamiaobao, Chia Tai Group, China) were uniformly managed. Shrimps (length, 5 mm) were cultured in 18 tanks with 800 individuals each and were randomly divided into three groups (two control groups and one treatment group) with six replicates. Commercial *Enterococcus faecium* powder (EF-P, number of live bacteria ≈ 100 × 10^8^ CFU/g, Yichun Strong Microbial Technology Co., Ltd., Jiangxi, China) was selected for the treatment group (Tre). The blank control (CK1) was provided normal feeds, while the medium control (CK2) was provided feeds mixed with sterilized de-Man Rogosa Sharpe (MRS) medium (MM-F, 5 mL of MRS medium per gram of feed). Tre was provided feeds mixed with activated commercial *E. faecium* powder (A-EF) at the same ratio three times a day. Detailed information on the activation, fermentation, and experimental setup are shown in Fig. 1.

The shrimps were starved for one day before sampling. After 51 days, we collected all shrimps and survival ratio (SR), unit yield (UY), specific growth rate (SGR), and feed conversion ratio (FCR) were estimated. Ten gut samples of large, medium, small shrimps including digesta were randomly collected as one biological replicate respectively to eliminate bias and a total of three biological replicates each tank was performed for DNA extraction. Importantly, there was no obvious disease or substantially abnormal death rates observed during cultivation management. Water temperature, dissolved oxygen (DO), oxidation–reduction potential (ORP), salinity and pH were measured in each tank every day with a probe (YSI 550A, USA). The concentrations of nitrite (NO_2_^−^), nitrate (NO_3_^−^), phosphate (PO_4_^3−^), and ammonium (NH_4_^+^) were analyzed according to standard methods (GB 17378.4-2007) at day 11, 41, and 51.

### DNA extraction and bacterial 16S rRNA gene amplification

The microbial DNA of shrimp gut, EF-P, and products of activation (A-EF) and fermentation (EF-F) were extracted according to the user’s manual of the QIAamp DNA Stool mini kit (Qiagen GmbH, Hilden, Germany). The concentrations of extracted DNA were measured on a NanoDrop ND-1000 spectrophotometer (NanoDrop Technologies, Wilmington, DE, USA). The bacterial primers 515F (5′–GTGCCAGCMGCCGCGGTAA–3′) and 806R (5′–GGACTACNNGGGTATCTAAT–3′) were used to amplify the V4 region of the 16S rRNA gene. Each sample was amplified in triplicate (50-μL reaction system) to reduce bias under the listed conditions: 25 cycles of denaturation at 95 °C for 30 s, annealing at 55 °C for 30 s, and extension at 72 °C for 45 s, with a final extension for 10 min. To check the expected amplification effect, PCR products were visualized on a 1.5% agarose gel. Triplicate amplicons were combined and purified by a PCR fragment purification kit (TaKaRa Biotech, Japan). The purified amplicons were quantified with a PicoGreen-iT dsDNA Assay Kit (Invitrogen, Carlsbad, CA, USA). Equal concentrations of amplicons were combined into one pooled sample and sequenced on a MiSeq PE 2 × 250 platform (Illumina, Majorbio, Shanghai, China).

### Processing of sequencing data

Raw data were processed with QIIME v1.9.1 pipeline [61]. Using the UCHIME algorithm, chimeric sequences were identified [62] and removed from subsequent analysis. Bacterial phylotypes were identified using UCLUST [63] and were classified into operational taxonomic units (OTUs) at a 97% similarity level. For each OTU, the most abundant sequence was regarded as the representative sequence and was then taxonomically assigned using the SILVA_128 database (https://www.arb-silva.de/documentation/release-128/). The matching results yielded 2,702,120 reads, and 26,002 reads were unclassified due to chimeras. Archaea and Chloroplast sequences were removed using “filter_taxa_from_otu_table.py”, as well as other sequences that could not be assigned to bacteria. Afterwards, singletons (n = 2) were also discarded. To correct for varying sampling efforts, the data were randomly rarefied at the minimum sequencing depth (24,000 sequences). After that, the sequencing of 16S rRNA genes generated 1,296,000 high-quality sequences and 7656 OTUs based on 97% similarity. Finally, OTUs in no less than two samples were selected for β-diversity and function analysis and 3553 OTUs were generated.

### Bioinformatic analysis

QIIME was used to calculate the α-diversity indexes, including observed species (Richness), Shannon index, and phylogenetic diversity (PD). Pielou’s evenness (Evenness) was estimated with the Vegan R package. Nonparametric test (Mann–Whitney U test for two groups and Krustkal-Wallis H test for more than three groups) were performed on all data using SPSS v.16.0. Significance levels were considered at α < 0.05 with 95% confidence interval. Based on Bray–Curtis distances, principal coordinates analysis (PCoA) was applied to analyze the difference in gut microbiota and two factors (treatment and survival ratio) as dependent variables that are explained by the ordination scores were added using “envfit” in Vegan R package. The “betadisper” in Vegan R package was used to check the assumption of heterogeneity in dispersions according to Bray–Curtis distance [64]. Pairwise analyses of similarity (ANOSIM) and permutational multivariate analysis of variance (PERMAVONA) were applied to separate and quantitatively estimate the effects of treatment and survival ratio on the composition of gut microbiota by using “anosim” and “adonis” in Vegan R package [65]. We expected bacterial population with wider niche breadth at the community level to be more metabolically flexible [66], and the analysis was conducted using the “niche.width” in spaa R package [67]. Functional profiling of bacterial taxa was carried out using the Tax4Fun2 R package [68]. Shannon index of by KEGG orthology (KO) was measured. Additionally, PCoA and PERMAVONA were used to estimate the effects of treatment and on bacterial function represented by KO matrix based on Bray-Curits distance. Linear regression models were applied to estimate for significant correlations between Shannon index or community distance (PCoA1) or and function dissimilarity (PCoA1). Analysis of composition of microbiomes (ANCOM) was applied to detect the differentially abundant OTUs in the groups based on treatment and survival ratio [69], and 44 OTUs were further identified by custom values, with significant changes in their relative abundances between SH and SL in each group. The Boruta R package was used to find the relevant OTUs that caused differences in the gut microbiota of six groups (treatment × survival ratio) [70]. To account for the total effects of probiotic bacteria (*E. faecium*) and relevant OTUs on both gut microbiota and shrimp survival, partial least squares path modeling (PLS-PM) was run using functions available in the plspm R package.

### Oligotyping analysis

The python-pipeline v.2.7 (available from http://oligotyping.org) was used for oligotyping that uses highly variable single-nucleotide polymorphisms to achieve strain-level resolutions. After the initial calculation of Shannon entropy (variability) across each nucleotide position with the analyze-entropy script, members of genus *Enterococcus* were done, and the process were supervised until each oligotype had converged [18]. Considering the small number of sequences, we kept those almost resulting in oligotypes with regards to the detailed parameters: c (2), s (1), A (0), M(0), which specified that a unique oligotype represented at least 1% of sequence reads, was detected in at least 1 samples, and showed all unique read.

### Network analysis

To reduce the complexity and increase the credibility of the data sets, OTUs in no less than one-third of the samples were selected to construct the whole network and subgroups. All possible pairwise Spearman’s rank correlations (*r*) between the selected OTUs were estimated using the Picante R package. Only extraordinarily robust (r > 0.8 or r <  − 0.8) and statistically significant (*P* < 0.001) correlations were incorporated into the network analyses. All the networks exhibited scale-free characteristics, indicating non-random network structures. Network visualization, node-level topological features (i.e., degree, betweenness, closeness centrality, and eigenvector centrality), and modular analysis were performed in Gephi v.0.8.2. Afterward, 1000 Erdös–Réyni random networks, assigned the identical number of nodes and edges as the real metacommunity network, were generated within the igraph R package [71], where the topology characteristics, including modularity, clustering coefficient, and average path length, of both real and random networks were calculated and compared.

## Supplementary Information

**Additional file 1:** Supplementary tables and figures.

**Additional file 2:** Ko pathways of cluster A.

**Additional file 3:** OTUs from major modules.

**Additional file 4:** Relevant OTUs.

## Data Availability

The raw sequencing files from this study are available from the NCBI Sequence Read Archive (SRA) under accession number PRJNA591260.

## Abbreviations

MRS: De-Man Rogosa Sharpe
UY: Unit yield
SR: Survival ratio
FCR: Feed conversion ratio
SGR: Specific growth rate
DO: Dissolved oxygen
DNA: Deoxyribonucleic acid
rRNA: Ribosomal ribonucleic acid
PCR: Polymerase chain reaction
OTU: Operational taxonomic unit
QIIME: Quantitative insights into microbial ecology
PCoA: Principal coordinate analysis
ANOSIM: Analysis of similarity
ANCOM: Analysis of composition of microbiomes
PLS-PM: Partial least squares path modeling
