# Supplementary material for: Enterococcus faecium are associated with the modification of gut microbiota and shrimp post-larvae survival
Source: Anim Microbiome. 2021 Dec 24;3:88. doi: 10.1186/s42523-021-00152-x (PMC8710032; doi:10.1186/s42523-021-00152-x)
Supplement: Supplementary file 1 — Additional file 1: Supplementary tables and figures. [file 42523_2021_152_MOESM1_ESM.docx]

*The Following Supplementary accompanies the manuscript*

***Enterococcus faecium* are associated with the modification of gut microbiota and shrimp post-larvae survival**

Shicong Du ^1, 2, 3^, Wei Chen ^2^, Zhiyuan Yao ^1, 4 *,^ Xiaolin Huang ^2, 5^, Chen Chen ^5^, Haipeng Guo ^1, 2^, and Demin Zhang ^1, 2 *^

^1^State Key Laboratory for Managing Biotic and Chemical Threats to the Quality and Safety of Agro-products, Ningbo University, Ningbo 315211, China

^2^School of Marine Sciences, Ningbo University, Ningbo 315211, China

^3^School of Energy and Environment, City University of Hong Kong, Hong Kong SAR, China

^4^School of Civil and Environmental Engineering, Ningbo University, Ningbo 315211, China

^5^Zhejiang Mariculture Research Institute, Wenzhou 325099, China

**Table S1** Survival ratio (SR), unit yield (UY), and specific growth ratio (SGR) of shrimp and the feed conversion ratio (FCR) for each tank after 51 days

| Treatment | Replicates | SR (%) | UY (g/cm3) | SGR | FCR |
| --- | --- | --- | --- | --- | --- |
| CK1 | 1 | 81.28 | 211.50 | 10.03 | 0.76 |
|  | 2 | 74.88 | 178.00 | 9.51 | 0.93 |
|  | 3 | 41.95 | 84.00 | 5.99 | 1.33 |
|  | 4 | 66.67 | 157.25 | 8.12 | 0.85 |
|  | 5 | 56.73 | 136.00 | 10.42 | 0.96 |
|  | 6 | 70.48 | 160.95 | 7.92 | 0.99 |
| CK2 | 1 | 53.77 | 129.00 | 8.25 | 1.00 |
|  | 2 | 81.86 | 190.00 | 8.19 | 0.88 |
|  | 3 | 89.1 | 193.00 | 7.83 | 0.86 |
|  | 4 | 19.71 | 39.75 | 6.63 | 1.55 |
|  | 5 | 49.04 | 115.75 | 7.38 | 1.19 |
|  | 6 | 58.45 | 133.00 | 7.67 | 1.04 |
| Tre | 1 | 82.74 | 229.50 | 9.51 | 0.70 |
|  | 2 | 67.29 | 168.00 | 7.29 | 0.88 |
|  | 3 | 88.6 | 192.50 | 7.17 | 0.74 |
|  | 4 | 92.75 | 199.50 | 9.13 | 0.81 |
|  | 5 | 89.27 | 185.25 | 9.76 | 0.92 |
|  | 6 | 85.31 | 201.25 | 9.46 | 0.85 |

**Table S2** Distribution of abundant (mean relative abundance of > 1%) genera in *Enterococcus faecium* powder (EF-P) in activated *E. faecium* (A-EF), fermented feeds

| Taxonomy (%) | EF-P | A-EF | Fermented Feeds | |
| --- | --- | --- | --- | --- |
|  |  |  | MM-F | EF-F |
| *Bacillus* | 55.02 (1.16) | 10.76 (3.68) | 3.01 (1.20) | 5.16 (0.32) |
| *Enterococcus* | 24.29 (0.47) | 87.42 (4.38) | 1.10 (0.96) | 10.85 (0.21) |
| *Klebsiella* | 8.18 (0.21) | NA | NA | NA |
| *Proteus* | 2.73 (0.04) | NA | NA | NA |
| *Bartonella* | NA | NA | 89.48 (2.57) | 78.15 (0.74) |
| Others | 9.79 (0.49) | 1.82 (0.72) | 6.41 (0.98) | 5.84 (0.60) |

Values are means (s.d., n=3).

NA refers to that there was no such genus or its average relative abundance less than 1% in the samples.

MF-F and EF-F represent feeds treated by MRS sterile medium and activated *E. faecium*, respectively.

**Table S3** The list of abundant (mean relative abundance of > 1%) phylum/genus in gut microbiota

| Phylum | Overall  (Mean (%) ± s.d.) | Treatment (Mean (%) ± s.d.) | | | SR(Mean (%) ± s.d.) | |
| --- | --- | --- | --- | --- | --- | --- |
|  |  | CK1 (n = 19) | CK2 (n = 17) | Tre (n = 18) | SL (n = 24) | SH (n = 30) |
| **Proteobacteria** | 33.34 ± 9.04 | 35.70 ± 10.04 a | 37.31 ± 6.33 a | 27.09 ± 6.87 b | 41.14 ± 5.55 a | 27.1 ± 11.73 b |
| **Bacteroidetes** | 28.85 ± 9.43 | 26.55 ± 9.61 b | 25.51 ± 8.69 b | 34.44 ± 7.63 a | 21.06 ± 6.48 b | 35.08 ± 12.46 a |
| Actinobacteria | 11.97 ± 4.47 | 10.77 ± 3.06 | 10.89 ± 2.83 | 14.26 ± 6.05 | 10.92 ± 2.86 | 12.82 ± 10.67 |
| Planctomycetes | 11.08 ± 5.19 | 10.75 ± 6.26 | 12.84 ± 5.42 | 9.78 ± 3.13 | 13.43 ± 6.31 a | 9.20 ± 6.14 b |
| Verrucomicrobia | 8.98 ± 6.53 | 8.09 ± 8.66 b | 7.85 ± 4.20 | 11.00 ± 5.50 a | 4.57 ± 2.99 b | 12.52 ± 12.93 a |
| Chloroflexi | 1.45 ± 1.98 | 2.33 ± 2.92 | 1.4 0± 1.23 a | 0.58 ± 0.31 b | 2.34 ± 2.7 a | 0.75 ± 1.01 b |
| Tenericutes | 1.26 ± 2.84 | 2.28 ± 4.45 a | 0.73 ± 0.85 | 0.69 ± 1.34 b | 2.32 ± 3.97 a | 0.42 ± 1.51 b |
| Saccharibacteria | 1.19 ± 1.30 | 1.31 ± 1.87 | 1.06 ± 1.11 | 1.18 ± 0.62 | 0.35 ± 0.20 b | 1.86 ± 2.84 a |
| Others | 1.87± 2.28 | 2.23 ± 2.17 a | 2.41 ± 2.64 a | 0.99 ± 1.83 b | 3.87 ± 2.09 a | 0.27 ± 0.28 b |

| Genus | Overall  (Mean (%) ± s.d.) | Treatment (Mean (%) ± s.d.) | | | SR (Mean (%) ± s.d.) | |
| --- | --- | --- | --- | --- | --- | --- |
|  |  | CK1 (n = 19) | CK2 (n = 17) | Tre (n = 18) | SL (n = 24) | SH (n = 30) |
| *Haloferula* | 5.96 ± 4.49 | 5.25 ± 6.20 b | 6.13 ± 3.06 ab | 6.56 ± 3.52 a | 3.84 ± 2.77 b | 7.66 ± 4.91 a |
| *Formosa* | 5.89 ± 6.14 | 5.57 ± 5.15 ab | 3.77 ± 5.24 b | 8.25 ± 7.29 a | 2.19 ± 2.53 b | 8.86 ± 6.58 a |
| ***Algoriphagus*** | 5.04 ± 3.45 | 3.50 ± 3.46 b | 4.52 ± 2.28 b | 7.15 ± 3.44 a | 2.15 ± 2.41 b | 7.34 ± 2.17 a |
| *Robiginitalea* | 4.37 ± 2.76 | 4.79 ± 3.34 | 4.16 ± 2.89 | 4.11 ± 1.95 | 3.85 ± 2.97 | 4.78 ± 2.56 |
| *Planctomyces* | 3.07 ± 2.11 | 3.43 ± 2.15 | 3.37 ± 2.60 | 2.43 ± 1.39 | 3.87 ± 2.40 a | 2.44 ± 1.62 b |
| *Pirellula* | 2.95 ± 1.30 | 2.89 ± 1.32 | 2.75 ± 1.34 | 3.21 ± 1.26 | 2.57 ± 1.39 b | 3.26 ± 1.15 a |
| *Haliea* | 2.43 ± 2.94 | 3.06 ± 3.90 | 2.54 ± 3.07 | 1.68 ± 0.90 | 3.73 ± 4.00 a | 1.40 ± 0.77 b |
| ***Vibrio*** | 2.17 ± 3.53 | 2.41 ± 3.26 a | 3.14 ± 4.08 a | 1.00 ± 3.08 b | 4.11 ± 3.79 a | 0.62 ± 2.40 b |
| *Mycobacterium* | 1.95 ± 1.78 | 2.74 ± 2.17 | 1.75 ± 1.69 | 1.31 ± 1.03 | 3.28 ± 1.91 a | 0.89 ± 0.53 b |
| *Rhodopirellula* | 1.58 ± 1.16 | 1.16 ± 0.87 b | 2.15 ± 1.36 a | 1.49 ± 1.06 ab | 2.28 ± 1.29 a | 1.03 ± 0.64 b |
| *Ruegeria* | 1.41 ± 1.16 | 1.96 ± 1.31 a | 1.35 ± 1.23 ab | 0.88 ± 0.58 b | 2.06 ± 1.40 a | 0.88 ± 0.53 b |
| *Blastopirellula* | 1.39 ± 0.83 | 1.09 ± 0.72 b | 1.91 ± 0.99 a | 1.23 ± 0.51 | 1.69 ± 1.03 | 1.16 ± 0.53 |
| *Winogradskyella* | 1.21 ± 1.84 | 2.07 ± 2.66 | 0.90 ± 0.97 | 0.60 ± 0.92 | 2.38 ± 2.28 a | 0.28 ± 0.24 b |
| *Candidatus Bacilloplasma* | 1.14 ± 2.79 | 2.09 ± 4.40 | 0.6 ± 0.81 | 0.64 ± 1.24 | 2.16 ± 3.92 a | 0.32 ± 0.68 b |
| *Nitrosomonas* | 1.12 ± 0.93 | 1.52 ± 1.29 ab | 1.19 ± 0.68 b | 0.63 ± 0.29 a | 1.87 ± 0.95 a | 0.52 ± 0.23 b |
| Others | 58.30 ± 7.89 | 56.46 ± 9.66 | 59.78 ± 6.07 | 58.84 ± 7.36 | 57.97 ± 9.49 | 58.56 ± 6.49 |

Different lower-case letters represent differences between two subgroups that were significant at *p* < 0.05 according to nonparametric Mann-Whitney U test with Monte Carlo (2-tailed) at 95% confidence interval.

Phyla or genera are differentially abundant both between Tre and the controls and between SH and SL are in bold.

**Table S4** Topological properties of the real co-occurrence network for whole gut microbiota and its associated random networks

|  | Real network | | | | | | | | Random networks | | | | |  |
| --- | --- | --- | --- | --- | --- | --- | --- | --- | --- | --- | --- | --- | --- | --- |
|  | | Nodes | Edges | Modularity | Average clustering coefficient | Network diameter | Average path length | Average degree | |  | Modularity (SD) | Average clustering coefficient (SD) | Average path length (SD) | |
| Whole | | 1,125 | 4,330 | 1.065 | 0.362 | 17 | 4.709 | 3.84 | |  | 0.267(0.006) | 0.006(0.001) | 3.670(0.003) | |

The number in () means s.d., n=1000

Table S5 Topological properties of co-occurrence network for subgroups

| Subgroup | Nodes | Edges | Modularity | Average clustering coefficient | Average path length | Average degree |
| --- | --- | --- | --- | --- | --- | --- |
| CK1 | 539 | 893 (72) | 0.902 | 0.582 | 6.619 | 2.59 |
| CK2 | 390 | 362 (15) | 1.016 | 0.836 | 1.322 | 1.663 |
| Tre | 613 | 1278 (8) | 0.638 | 0.502 | 8.416 | 3.822 |
| SL | 433 | 367 (16) | 0.993 | 0.378 | 2.87 | 1.367 |
| SH | 217 | 183 (11) | 0.974 | 0.369 | 2.093 | 1.253 |

The number in () represents negative edges

**Table S6** List of predicted pathway in Cluster A

| Pathway | Level 1 | Level 2 | Level 3 |
| --- | --- | --- | --- |
| ko04213 | Longevity regulating pathway - multiple species | Aging | Organismal Systems |
| ko04113 | Meiosis - yeast | Cell growth and death | Cellular Processes |
| ko00130 | Ubiquinone and other terpenoid-quinone biosynthesis | Metabolism of cofactors and vitamins | Metabolism |
| ko05231 | Choline metabolism in cancer | Cancers: Overview | Human Diseases |
| ko04072 | Phospholipase D signalling pathway | Signal transduction | Environmental Information Processing |
| ko00332 | Carbapenem biosynthesis | Biosynthesis of other secondary metabolites | Metabolism |
| ko00720 | Carbon fixation pathways in prokaryotes | Energy metabolism | Metabolism |
| ko00290 | Valine, leucine and isoleucine biosynthesis | Amino acid metabolism | Metabolism |
| ko04964 | Proximal tubule bicarbonate reclamation | Excretory system | Organismal Systems |
| ko00965 | Betalain biosynthesis | Biosynthesis of other secondary metabolites | Metabolism |
| ko00253 | Tetracycline biosynthesis | Metabolism of terpenoids and polyketides | Metabolism |
| ko04728 | Dopaminergic synapse | Nervous system | Organismal Systems |
| ko05034 | Alcoholism | Substance dependence | Human Diseases |
| ko04726 | Serotonergic synapse | Nervous system | Organismal Systems |
| ko05030 | Cocaine addiction | Substance dependence | Human Diseases |
| ko05031 | Amphetamine addiction | Substance dependence | Human Diseases |
| ko04138 | Autophagy - yeast | Transport and catabolism | Cellular Processes |
| ko00901 | Indole alkaloid biosynthesis | Biosynthesis of other secondary metabolites | Metabolism |
| ko01051 | Biosynthesis of ansamycins | Metabolism of terpenoids and polyketides | Metabolism |
| ko00340 | Histidine metabolism | Amino acid metabolism | Metabolism |
| ko05418 | Fluid shear stress and atherosclerosis | Cardiovascular diseases | Human Diseases |
| ko00040 | Pentose and glucuronate interconversions | Carbohydrate metabolism | Metabolism |
| ko04016 | MAPK signaling pathway- plant | Signal transduction | Environmental Information Processing |
| ko00525 | Acarbose and validamycin biosynthesis | Biosynthesis of other secondary metabolites | Metabolism |
| ko01055 | Biosynthesis of vancomycin group antibiotics | Metabolism of terpenoids and polyketides | Metabolism |
| ko00910 | Nitrogen metabolism | Energy metabolism | Metabolism |
| ko05100 | Bacterial invasion of epithelial cells | Infectious diseases: Bacterial | Human Diseases |

**Fig. S1** Dynamics of environmental factors over sampling days. DO: dissolved oxygen; ORP: oxidative redox potential.

**
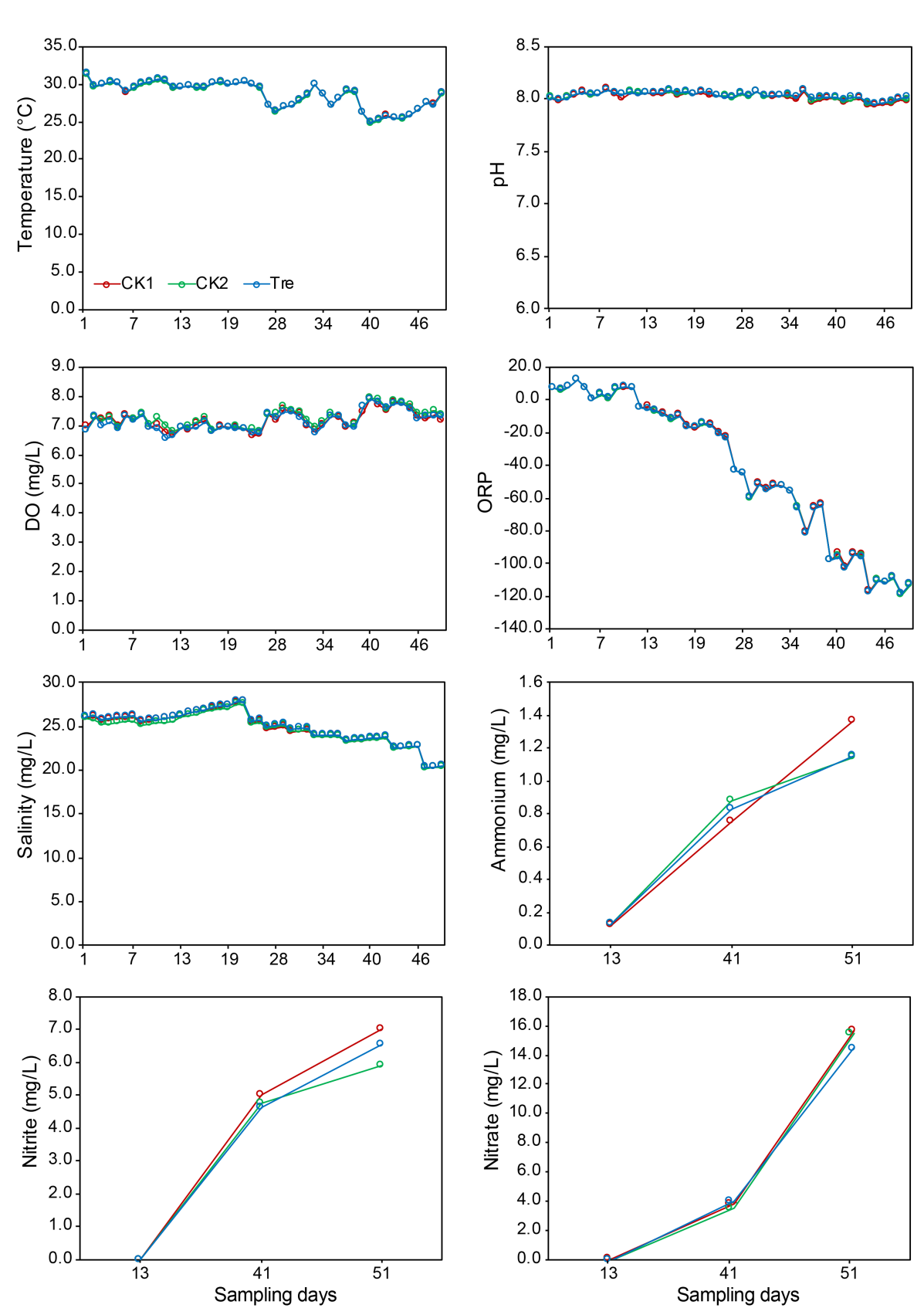
**

**Fig. S2** Shrimp survival ratios (SR) across three subgroups for treatment. Dashed circles mean SR in these tanks less than 70%, solid circles mean SR in these tanks more than 70%

**Fig. S3** Oligotype types of *Enterococcus* found in shrimp gut samples and samples related to *E. faecium* powder (EF-P).

**
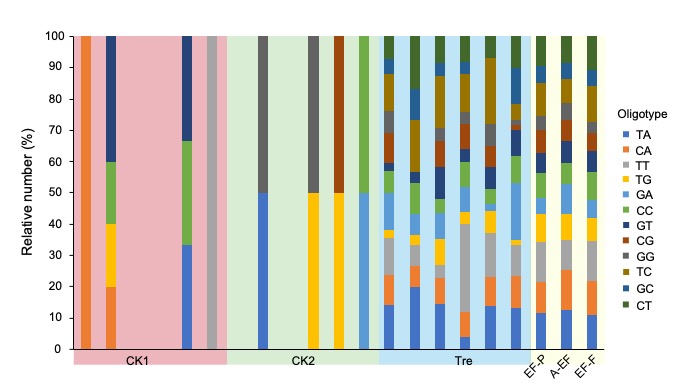
**

**Fig. S4** The reads distribution of OTU 1462 across samples

**
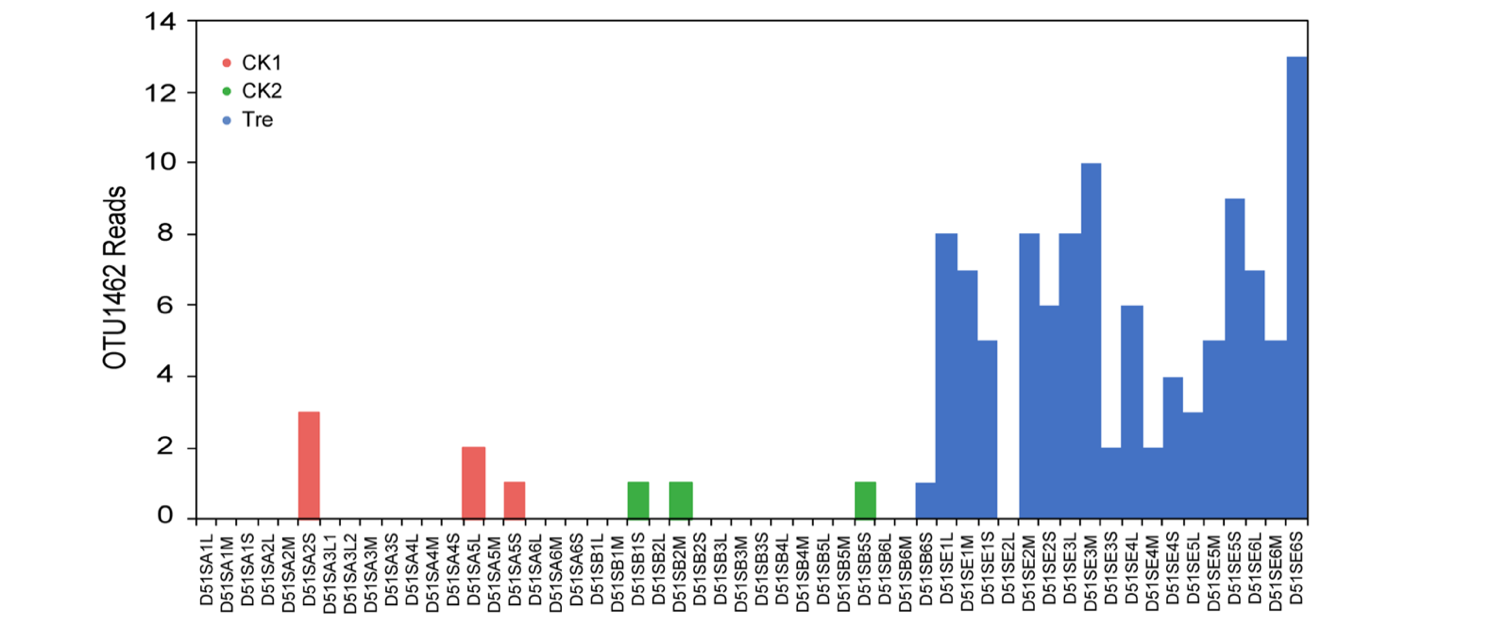
**

**Fig. S5** Comparison of mean niche breadths in all OTUs for treatment (A) and SR (B) (*** *p* < 0.001; Mann-Whitney U test with Monte Carlo (2-tailed))

**Fig. S6** Heatmap of abundant (mean relative abundance of > 1%) phyla (A) and genera (B) in gut microbiota. As “Others” in the description of genera accounted more than 50% relative abundance, so we did not include it in the follow

**Fig. S7** Changes in the abundance of gut microbial taxa for treatment. The significantly more abundant taxa in Tre are shown in the right panel, while the significantly less abundant taxa in Tre on the left

**Fig. S8** The co-occurrence patterns in gut microbiota. (A) Metacommunity co-occurrence network of OTUs. The nodes are colored according to the different types of modularity class (left) and phylum (right), respectively. The size of each node is proportional to the relative abundance. Major modules have more than 80 nodes, and other modules include all small modules (n = 126) with nodes ≤ 45 per module. (B) Ternary plots showing relative abundance of OTUs from modules I–VI in the three groups. Each circle represents one individual OTU. For each OTU, abundance was averaged over all samples at each group.

**Fig. S9** Unique node-level topological features of different OTUs with degree, betweenness centrality, closeness centrality, and eigenvector centrality for treatment (A) and SR (B) (****p* < 0.001; Mann-Whitney U test with Monte Carlo (2-tailed))
